# Pharmacy customers’ views towards the potential introduction of pharmacist prescribing: A survey study

**DOI:** 10.1371/journal.pone.0325208

**Published:** 2025-05-29

**Authors:** Jan Siefert, Niklas Zimmermann, Kandarp Thakkar, Ágnes Mészáros

**Affiliations:** 1 Department of Pharmacy Administration, University Pharmacy, Semmelweis University, Budapest, Hungary; 2 Department of Pharmacy, University Hospitals Plymouth National Health Service Trust, Plymouth, United Kingdom; Endeavour College of Natural Health, AUSTRALIA

## Abstract

**Background:**

Pharmacist prescribing has been introduced in several countries owing to systemic issues within healthcare. This systematic change can improve general public’s access to prescribed medication. In Germany, pharmacist prescribing and its acceptance among the population remains unexplored because legislators insist on the exclusive right of doctors to prescribe medication.

**Objectives:**

This study investigated pharmacy customers’ views towards pharmacist prescribing. The study sought to determine whether pharmacy customers in Germany could imagine the introduction of pharmacist prescribing.

**Methods:**

Pharmacy customers in Germany participated in an anonymised survey in August–October 2023. Participants were recruited using a convenience sampling method, mainly from community pharmacies. The questionnaire comprised 28 questions and covers participants’ characteristics, key aspects of patient care and statements regarding the topic of pharmacists prescribing. The collected data were coded and analysed using IBM SPSS version 28.0.1.

**Results:**

In total, 316 participants completed the survey. The average age was 51.0 years, and the majority were females (69.6%, n = 220). Overall, participants reported a high level of satisfaction with the general services provided by community pharmacies. A high to very high level of trust in their community pharmacist (CP) was reported by 84.4% of the participants. A total of 79.1% of the participants stated that pharmacists should be trained for prescribing authorisations. A key finding of this research was that most participants (88.0%), regardless of age and gender, agreed that the concept of pharmacist prescribing is favourable.

**Conclusion:**

Participants across all age groups were generally positive about pharmacist prescribing and supportive of its implementation, while expressing the need for pharmacists to undergo further training tailored to the specific prescribing authorisation. This study provides groundwork for further research and contributes to policy-making considerations.

## Introduction

High-quality, safe patient care focuses on prevention, diagnosis, therapy and disease management, alongside overall well-being [1]. This care is delivered by various healthcare professionals, either independently or supervising non-professionals [2].

Ambulatory care in Germany is predominantly delivered by self-employed general practitioners (GPs) in private practices. Although these practices operate independently, they are a part of the statutory health insurance system, covering approximately 90% of the German population and ensuring access to outpatient services via participating physicians [3]. In 2021, approximately 60,601 GPs were working within this system, representing a ratio of approximately one GP per 1,373 inhabitants [4]. However, the distribution of GP services is not uniform, with rural regions facing substantial shortages [5]. Projections suggest that by 2035, approximately 11,000 GP positions will remain vacant, leaving approximately 40% of districts experiencing or at risk of inadequate GP provision [5]. This problem is further exacerbated by the ageing workforce, with 68.5% of GPs being >50 years in 2023 [6].

In Germany, community pharmacies are privately owned because pharmacy chains are prohibited. Each pharmacist can own a maximum of four pharmacies, ensuring a relatively dense distribution throughout urban and rural areas. On average, there is one pharmacy for every 4,761 inhabitants [7].

In many countries, including Germany, the demographic change in the population, reflected in age pyramid reversal, has constantly evolved the approach for ensuring best possible care for patients. Considering an ageing population, healthcare systems are facing new challenges [8,9]. Innovative approaches and practise-oriented solutions are needed for accessible, affordable and high-quality comprehensive patient care, while avoiding overburdening [10].

The extension of the prescribing authority to other health care professionals via non-medical prescribing is a promising approach. Country-specific systems for non-medical prescribing initially started with nurses being authorised to prescribe medication within a limited scope [11]. The success of such systems assign prescribing duties to healthcare professionals; this pivotal moment marked the beginning of pharmacist prescribing — pharmacists started filling the role of prescribing medications that was exclusively and historically held by physicians. Pharmacist prescribing is already practised in countries such as the United Kingdom (UK), Canada, the USA, Australia and Poland [12–17]. However, the extent of pharmacists’ prescribing powers considerably varies depending on the country and region.

The UK, for example, has established two different models of pharmacist prescribing. The first model — the supplementary prescribing model, previously named dependent prescribing — was introduced in 2003 and refers to the collaboration between an independent prescriber and an additional prescriber who could be a pharmacist [15,18,19]. The primary responsibility of diagnosis lies with the doctor, whereas the pharmacist is responsible for monitoring and continuing the therapy within a clinical management plan [20]. The second model is the independent prescribing model, which was introduced in 2006, in which independent prescribers were healthcare professionals who could autonomously and independently prescribe prescription drugs or therapies [12,21–23].

In Poland, community pharmacists (CPs) have been authorised to prescribe medication in emergencies since 2002. From 2020 onwards, the prescribing authority of Polish pharmacists was extended to consider prescribing medicines within the medical therapies initiated by a doctor. Therefore, the most common reason for prescribing medication is to issue a repeat prescription if the patient cannot obtain a prescription from their doctor. The potential reimbursement by the health insurance funds for medication prescribed by the pharmacist is only granted if the pharmacist prescribes medication for self or family members [12,24].

At present, no pharmacist prescribing exists in Germany. Beyond the pharmacist prescribing models established in other countries, a new and alternative model has been developed herein. A potential model for implementation in Germany could involve pharmacists being authorised to issue repeat prescriptions when a physician has issued three prescriptions for the medication in question, given a clinical management plan is established. ‘A repeat prescription is a prescription for a medicine that you have taken before or that you use regularly’ [25]. Under this model, the clinical management plan would delineate the conditions under which the patient and physician consent to the pharmacist’s authority to prescribe medication, specifying the long-term therapy medications for which the pharmacist may issue future prescriptions.

Other models work on the possibility of a CP being allowed to make diagnoses for minor illnesses and prescribe medication [26]. An example of such a model is the ‘Pharmacy First’ programme by NHS England in early 2024, which gives CPs the possibility to offer prescription medicines to patients for treating seven common conditions, including uncomplicated urinary tract infections and acute otitis media. Thus, patients do not need an appointment with their GP beforehand [27,28].

Reportedly, introducing models of pharmacist prescribing can reduce the challenges in healthcare systems [29]. Pharmacists’ prescribing services are generally seen as easily accessible [29] and more accessible than other healthcare professionals, which could make them the primary healthcare provider for patients [12]. Therefore, pharmacist prescribing improves access to medicines while reducing unnecessary emergency room visits and burden on primary care providers [12,30]. Tinelli et al reportedly that 79% of participants would take their medication if prescribed by a pharmacist [31]. Waszyk-Nowaczyk et al. showed that interdisciplinary collaboration between professional groups significantly influenced complex treatments and provided numerous opportunities for improved health outcomes [32]. Both sides provide valuable insights and recommendations, leading to a holistic care plan.

Pharmacist prescribing, in addition to the associated benefits observed in other countries, has the potential to enhance patient care in Germany and improve the structure of the German healthcare system. Similar to several countries, there is a shortage of GPs in Germany, especially in rural areas, leading to difficulties for patients to connect with doctors for obtaining repeated prescriptions [5]. In Germany, the clear division of roles between doctors and pharmacists continues, implying that prescribing by pharmacists is neither practised, nor has any plans for its introduction. Moreover, the absence of publications has led to a notable knowledge gap in the German healthcare landscape, reflecting a lack of research on the subject. This observation was made during a review of the literature. This study aimed to investigate pharmacy customers’ views towards pharmacist prescribing and its potential implementation in Germany.

## Methods

A cross-sectional survey study was conducted using a hybrid methodology, combining offline and online data collection techniques, targeting pharmacy customers aged ≥18 years who visited community pharmacies. The recruitment and data collection period started on 1 August 2023 and ended on 31 October 2023. The questionnaire was formulated for the German healthcare system and encompassed various topics concerning pharmacist prescribing.

### Questionnaire development

During the initial stage, a pilot study (n = 31) was conducted using a paper-based version of the questionnaire. Participants were recruited via convenience sampling from five community pharmacies. The questionnaire was assessed in these settings to identify any potential problems with clarity, structure and wording. Adjustments were made based on the feedback from participants, followed by minimal modifications in the word choice and sequence of questions. To ensure face and content validities, the instrument was reviewed separately by the study authors and further evaluated by two experts from Semmelweis University.

The final questionnaire contained 28 questions, beginning with items on pharmacy customers’ demographics, including age, gender and the number of prescription drugs they took permanently. The second part gathered information on key aspects of patient care, including questions and statements on trust in CPs and GPs, satisfaction with access and opening hours of community pharmacies and GP practices, satisfaction with the CP advisory skills and interruptions in long-term drug therapies because of few repeat prescriptions. Participants’ responses were assessed using closed response options, including five-point Likert and rating scales. The third part of the questionnaire contained attitudinal statements about pharmacist prescribing. Participants’ level of agreement with these statements was assessed and quantified using five-point Likert scale response options (ranging from ‘Do not agree at all’ to ‘Fully agree’).

To complete and return the questionnaire, participants had to select an answer for each question. For all questions, the option ‘no answer’ was provided and deemed an acceptable response for successfully completing the questionnaire. The responses were recorded anonymously, without collecting any personal data or IP addresses [33]. Questions regarding the participant’s characteristics were designed to prevent personal identification, ensuring anonymity and confidentiality. The LimeSurvey platform provides extensive options for ensuring data protection and anonymity. Data protection standards for EU users were fully adhered per the General Data Protection Regulation (GDPR) [34].

### Data collection and recruitment

To maximise the number of participants, the survey was administered via various channels. Participants were recruited using a convenience sampling approach, primarily from five community pharmacies in the federal state of Baden-Württemberg, including one in an urban area and four in rural areas. In each pharmacy, a dedicated stand was placed in a less central area and remained accessible throughout recruitment. This allowed customers to find out about the study and take part at any point during the pharmacy opening hours. The stand featured a study poster, an iPad™ and flyers containing a QR code that linked to the online survey. Participants had several options for completing the survey: they could complete the questionnaire on-site using the provided iPad™ or their smartphone by scanning the QR code. Alternatively, they could fill out a printed questionnaire on-site or take it home, complete it and return it to the pharmacy at a later time. Moreover, the QR code included on the flyers enabled participants to access and complete the survey online at their convenience, from any location. Pharmacy customers and patients were recruited using various methods, including self-enrolment at the designated stand. Pharmacy staff actively drew all customers’ attention to the study for 1 h each day, Monday to Saturday, and encouraged them to visit the designated stand for more details. The specific hour varied each day to ensure a fair representation of all times throughout recruitment. To further extend the survey’s reach, it was shared on the public Facebook© pages of the five community pharmacies involved in the study, resulting in an undefined sampling frame. Consequently, the actual sample size and response rate could not be determined.

### Statistical analysis

The modification of the questionnaire after the pilot study potentially influenced the responses and thus were excluded from the final analysis. Data from the completed questionnaires were coded and analysed using the software package for statistical analyses 28.0.1 (IBM SPSS). In addition to the descriptive statistical analysis, 95% confidence intervals were calculated to determine the strength of the associations between nominal variables. The response options were grouped into categories wherever appropriate for the analysis. For instance, to assess a negative attitude, the Likert scale responses ‘Somewhat disagree’ and ‘Do not agree at all’ were combined as one category. The Mann-Whitney U test, as a non-parametric test, was used in several subgroup analyses to determine whether there were statistically significant differences between the distributions of two independent samples. A p-value of ≤0.05 was used as the cut-off value for statistical significance.

### Ethical considerations

Participants were asked to provide informed consent on the first page of the survey. This page outlined the purpose of the study, any potential risks and benefits, alternatives to participation, their right to withdraw at any time and where to find further information. Voluntary and informed consent was given electronically via the LimeSurvey platform when participants selected ‘yes’, which then allowed them to proceed to the main survey questions. Ethics committee approval was waived in June 2023 due to the circumstances outlined in the ethics evaluation document.

## Results

A total of 316 participants completed the questionnaire by the end of the survey period. The demographic characteristics of the participants are shown in Table 1. The largest group of participants, 28.5% (n = 90) belonged to the age group of 50–59 years. The age distribution of the study participants was compared with that of the general population of Baden- Württemberg aged ≥18 years [35]. Although the general population exhibited a typical age distribution, the sample revealed an underrepresentation of the 30–39, 40–49 and ≥70 years age groups, in addition to an overrepresentation of the 50–59 age group. The age groups 18–29 and 60–69 years were proportional to the overall population. The average age of the participants in our study, 51.04 years, closely aligned with that of 50.75 years for individuals aged ≥18 years in Baden-Württemberg. The gender distribution in this study sample showed that 69.6% (n = 220) of the participants were female. This distribution indicated an overrepresentation of women compared with the overall population [35]. More than three-quarters of participants took either no prescription medication (39.6%; n = 125) or one or two prescription medications (38.9%; n = 123) for a long-term condition. The group with five or more prescription drugs, predominantly defined as polypharmacy patients [36], was the smallest group, with 7.9% (n = 25).

**Table 1:** Participant characteristics (N = 316).

Characteristics	Number of participants (%)	Population Data* (%)
*Age (years)*		
18–29	55 (17.4)	1,584,380 (17.0)
30–39	37 (11.7)	1,509,733 (16.2)
40–49	32 (10.1)	1,373,919 (14.7)
50–59	90 (28.5)	1,697,926 (18.2)
60–69	58 (18.4)	1,447,430 (15.5)
≥70	43 (13.6)	1,711,786 (18.4)
No answer	1 (0.3)	
*Gender*		
Male	94 (29.7)	4,591,708 (49.2)
Female	220 (69.6)	4,591,708 (50.8)
Diverse	1 (0.3)	
No answer	1 (0.3)	
*How many prescription drugs do you take regularly?*		
None	125 (39.6)	
One or two	123 (38.9)	
Three or four	43 (13.6)	
Five or more	25 (7.9)	

*The population data were obtained from the Baden-Württemberg State Statistical Office, based on the 2022 census [35].

The second part of the questionnaire focused on the key aspects of patient care, such as communication, accessibility, satisfaction and trust. These questions centred around GP practices and community pharmacies, along with GPs and pharmacists as service providers.

Participants’ trust towards their CP was rated as high to very high in 84.4% (n = 267) of cases, whereas trust in GPs was rated as high to very high in 73.8% (n = 233) of cases. The answers are summarised in Table 2.

**Table 2:** Trust in healthcare professionals.

Trust	Number of responses (%)					
	Very low	Low	Middle	High	Very high	No answer
How much trust do you have in the pharmacists at your community pharmacy?	1 (0.3)	4 (1.3)	41 (13.0)	94 (29.7)	173 (54.7)	3 (0.9)
How much trust do you have in your general practitioner (GP)?	4 (1.3)	7 (2.2)	66 (20.9)	125 (39.6)	108 (34.2)	6 (1.9)

Most (90.5%, n = 286) participants reported being somewhat satisfied or fully and completely satisfied with the advisory skills of their CP (Table 3).

**Table 3:** Satisfaction.

	Number of responses (%)					
	Not at all satisfied	Somewhat unsatisfied	Neither satisfied nor dissatisfied	Somewhat satisfied	Fully and completely satisfied	No answer
How satisfied are you with the accessibility of your community pharmacy?	0 (0.0)	5 (1.6)	11 (3.5)	58 (18.4)	235 (74.4)	7 (2.2)
How satisfied are you with the accessibility of your general practitioner (GP)?	9 (2.8)	50 (15.8)	39 (12.3)	111 (35.1)	102 (32.3)	5 (1.6)
How satisfied are you with the advisory skills of your community pharmacists?	0 (0.0)	11 (3.5)	17 (5.4)	75 (23.7)	211 (66.8)	2 (0.6)
	**Do not agree at all**	**Somewhat disagree**	**Neutral**	**Somewhat agree**	**Fully agree**	**No answer**
I am satisfied with the waiting times at my GP practise. (It is about the time I have to wait before the actual appointment).	24 (7.6)	70 (22.2)	85 (26.9)	80 (25.3)	51 (16.1)	6 (1.9)
I am satisfied with the opening hours of my GP practise.	22 (7.0)	51 (16.1)	69 (21.8)	105 (33.2)	64 (20.3)	5 (1.6)
I am satisfied with the opening hours of my community pharmacy.	4 (1.3)	7 (2.2)	33 (10.4)	89 (28.2)	180 (57.0)	3 (0.9)

Regarding participants’ satisfaction with the accessibility of their community pharmacy, 92.8% (n = 293) of participants reported being somewhat satisfied or completely satisfied, whereas 1.6% (n = 5) reported being somewhat dissatisfied or not satisfied at all. Participants who were either somewhat satisfied or fully and completely satisfied with their GP’s accessibility were markedly low at 67.4% (n = 213) (Table 3).

Over half of all participants (53.5%; n = 169) were satisfied with the opening hours of their GP practise, whereas only 41.5% (n = 131) of participants were satisfied with the waiting times before the actual doctor’s appointment. Satisfaction with the opening hours of the community pharmacy was reported by 85.1% (n = 269) of the participants (Table 3).

The frequency with which participants ran out of medication for their long-term therapy because of not receiving a repeat prescription in time is presented in Table 4. Most participants (66.8%; n = 211) stated that this situation had never or only rarely occurred, whereas 15.8% (n = 50) reported that it had occurred occasionally. Only 6.3% (n = 20) reported that it had occurred frequently or very frequently.

**Table 4:** Interruptions in long-term drug therapies because of lack of repeat prescriptions.

	Number of responses (%)					
	Never	Occurred Rarely	Occurred Occasionally	Frequently	Occurred very frequently	No answer
Please rate how often you have already run out of medication for your long-term therapy because you did not receive a repeat prescription on time to continue the therapy without interruption.	147 (46.5)	64 (20.3)	50 (15.8)	15 (4.7)	5 (1.6)	35 (11.1)

The next section included various attitudinal statements on pharmacist prescribing. The statements and participants’ levels of agreement are presented in Table 5.

**Table 5:** Participants’ opinions on pharmacist prescribing.

	Number of responses (%)					
	Do not agree at all	Somewhat disagree	Neutral	Somewhat agree	Fully agree	No answer
Pharmacists being responsible for the clinical management of a disease previously diagnosed by a doctor and allowed to prescribe medication within their area of responsibility is a good thing. (Mainly refers to the issuing of prescriptions for drugs for long-term therapy [repeat prescriptions]).	7 (2.2)	16 (5.1)	31 (9.8)	104 (32.9)	154 (48.7)	4 (1.3)
I would trust my community pharmacist with diagnosis and prescription for minor illnesses.	10 (3.2)	18 (5.7)	25 (7.9)	107 (33.9)	153 (48.4)	3 (0.9)
If pharmacists are allowed to issue repeat prescriptions, I would prefer to have the repeat prescription issued by my community pharmacist.	6 (1.9)	8 (2.5)	18 (5.7)	77 (24.4)	202 (63.9)	5 (1.6)
I am confident that my prescribing community pharmacist would prescribe as safely as my GP.	10 (3.2)	14 (4.4)	40 (12.7)	100 (31.6)	150 (47.5)	2 (0.6)
The clinical assessment skills of pharmacists should be expanded before they are allowed to prescribe medicines.	9 (2.8)	28 (8.9)	96 (30.4)	88 (27.8)	84 (26.6)	11 (3.5)
I consider it a basic prerequisite for pharmacists to undergo further training per their prescribing authorisation.	4 (1.3)	9 (2.8)	44 (13.9)	90 (28.5)	160 (50.6)	9 (2.8)
I would find it difficult to entrust sensitive and confidential matters to the pharmacist prescriber. (Even if the consultation took place in a separate room).	73 (23.1)	104 (32.9)	64 (20.3)	43 (13.6)	29 (9.2)	3 (0.9)
I am more interested in the quality of the consultation than in the profession of the person. (Concerning prescribing pharmacists or doctors).	7 (2.2)	8 (2.5)	96 (30.4)	92 (29.1)	99 (31.3)	14 (4.4)
I can imagine that my prescribing community pharmacist would be my first point of contact because it is more convenient to visit them than other service providers.	10 (3.2)	35 (11.1)	50 (15.8)	99 (31.3)	119 (37.7)	3 (0.9)
Using the nationwide community pharmacy network and the convenience of pharmacy location, access to prescriptions for minor illnesses or repeat prescriptions could be facilitated by prescribing community pharmacists.	6 (1.9)	6 (1.9)	29 (9.2)	100 (31.6)	167 (52.8)	8 (2.5)
It would be easier and quicker to make an appointment with my community pharmacist than with my GP.	8 (2.5)	24 (7.6)	67 (21.2)	77 (24.4)	129 (40.8)	11 (3.5)
Introducing pharmacist prescribing could relieve the burden on GP practices. (More time and resources for in-depth medical care of patients would be available in GP practices).	4 (1.3)	9 (2.8)	26 (8.2)	101 (32.0)	170 (53.8)	6 (1.9)
My health insurance company should cover the costs of medication prescribed by the pharmacists. (As is already the case with the existing drug supply).	2 (0.6)	2 (0.6)	9 (2.8)	55 (17.4)	244 (77.2)	4 (1.3)
Introducing pharmacist prescribing would save money in healthcare.	7 (2.2)	10 (3.2)	54 (17.1)	85 (26.9)	144 (45.6)	16 (5.1)
I am confident that the waiting times at my prescribing community pharmacist would be satisfactory. (It is about the time I have to wait before the actual appointment).	3 (0.9)	11 (3.5)	64 (20.3)	97 (30.7)	132 (41.8)	9 (2.8)
I think the concept of pharmacist prescribing is good, and I can imagine it being introduced in Germany on a certain scale.	8 (2.5)	4 (1.3)	23 (7.3)	101 (32.0)	177 (56.0)	3 (0.9)

A large proportion of participants (82.3%; n = 260) indicated that they would trust their CP for diagnosis and prescription for minor illnesses. Among the participants, 79.1% (n = 250) were confident that their CP would prescribe as safely as their GP. The model in which pharmacists are responsible for the clinical management of a disease previously diagnosed by a doctor and are therefore authorised to prescribe medication within their area of responsibility was positively received by 81.6% (n = 258). Furthermore, 88.3% (n = 279) of the participants stated that if pharmacists were allowed to issue repeat prescriptions, they would have the repeat prescription issued by their CP.

Most participants expressed a preference for pharmacists to enhance their clinical assessment skills before being authorised to prescribe medications. Therefore, 79.1% of the participants considered it a basic prerequisite for pharmacists to undergo further training in line with their prescribing authorisation. A subgroup analysis between men and women showed that both agreed to training, with no statistically significant difference between the groups (p > 0.05).

Most participants responded in the affirmative to the statement ‘I am more interested in the quality of the consultation as such than in the profession’. Concerning ‘I can imagine that my prescribing CP would be my first point of contact because it is more convenient to visit them than other service providers’, 69% (n = 218) of participants selected somewhat or fully agree. The level of agreement was consistent across all age groups because no statistically significant difference (p > 0.05) was observed between the age groups. Several participants (84.4%; n = 267) agreed that prescribing CPs could facilitate access to repeat prescriptions using the nationwide community pharmacy network and the convenience of the pharmacy location (proximity to work or home). In addition, 85.8% (n = 271) agreed that ‘Introducing pharmacist prescribing could relieve the burden on GP practices. (More time and resources for in-depth medical care of patients would be available in GP practices)’.

The concept of pharmacist prescribing was viewed by 72.5% (n = 229) of participants as a beneficial method for reducing healthcare costs. Almost all (94.6%; n = 299) agreed that health insurance companies should cover the costs of medicines prescribed by pharmacists.

The statement ‘I think the concept of pharmacist prescribing is good and I can imagine it being introduced in Germany on a certain scale’ was agreed upon by 88% (n = 278) of the participants. A subgroup analysis revealed no statistically significant differences (p > 0.05) for this statement between age groups or between men and women regarding their attitudes towards the concept of pharmacist prescribing. A further subgroup analysis was conducted to investigate whether participants taking one or two prescription drugs regularly viewed pharmacists prescribing differently from those taking five or more prescription drugs. The results showed that participants taking five or more prescription drugs regularly significantly agreed with pharmacist prescribing than those taking only one or two prescription drugs regularly (z = [−2.621], p = [0.009]). A Mann-Whitney U test was performed to evaluate whether participants who do not take any prescription drugs regularly assessed pharmacist prescribing differently from those who took five or more prescription drugs regularly. The results indicated that participants who took five or more prescription drugs regularly significantly agreed with pharmacist prescribing than those who did not take any prescription drugs regularly (z = [−2.608], p = [.009]).

## Discussion

This research represents the first investigation into the potential introduction of pharmacist prescribing in Germany from a pharmacy customers’ perspective. This study was intended to provide novel insights and understanding to examine an initial, baseline picture of pharmacy customers’ opinions in Germany on this subject. Simultaneously, it was expected to serve as a basis for future research projects in this area.

This study analysed key aspects of daily patient care, and the results showed that most participants were highly satisfied with the opening hours and accessibility of their community pharmacy and with the advisory skills of their CPs. Furthermore, several participants had a high to very high level of trust in their CPs. The annual representative surveys conducted by the Federal Association of Pharmaceutical Manufacturers (BAH) in Germany provided a similar picture regarding the public’s trust in pharmacists. The development of the BAH survey results over the last few years showed that German pharmacists enjoy a very high level of trust, and this trust has improved over the last few years. Reportedly, in Germany, CPs were the stakeholders in the healthcare system who enjoyed the most trust among the population [37]. In 2023, 78% of the German population reported having a high to very high level of trust in CPs [37]. Considerably, the overall high level of trust in pharmacists, combined with the generally high level of satisfaction with pharmacists’ advisory skills, may contribute to participants entrusting pharmacists with new roles, such as the authority to prescribe medicines. Thus, the statement ‘I am confident that my prescribing CP would prescribe as safely as my GP’, with which 79.1% of participants agreed, can be an evidence of the level of trust in CPs. Further research is required to determine the reasons for this concern.

In terms of potential models of pharmacist prescribing that could be implemented in Germany, 82.3% of participants agreed that they would trust their pharmacist to diagnose minor ailments and prescribe medication. In addition, most participants (81.6%) believed that a model in which pharmacists are responsible for the clinical management of a disease previously diagnosed by a doctor and are allowed to prescribe medicines within their area of responsibility is a good thing. Clinical management in this model primarily refers to issuing repeat prescriptions.

Considering the results of this research, participants seem aware of the potential benefits of introducing prescription pharmacists for minor illnesses or for repeat prescriptions. Reportedly, 69% of the participants stated that the pharmacist prescribing would be their first point of contact. Mansell et al. [38] reached a similar conclusion in their Canadian patient survey, examining the symptomatic course of minor ailments after treatment by a pharmacist prescribing. Patients reportedly chose their pharmacist prescriber to treat their minor ailments because of the convenience and their trust on the pharmacist. Most participants felt that making an appointment with their pharmacist prescriber would be easy and quick; this aligns with the finding that 88.3% of these participants expressed a preference for having their repeat prescriptions issued by their CP, if pharmacists were allowed. Moreover, this assessment of participants was confirmed by studies from other countries, in which pharmacists were already authorised to prescribe. Reportedly, pharmacists were more readily accessible than other people in the healthcare system [12,39]. A recent scoping review examined the contribution of pharmacist independent prescribers to primary care. The authors highlighted several of their involvement at all levels of healthcare, including improved access to primary care, reduced delays in treatment, a lighter workload for physicians and an enhanced quality of life for patients. Remarkably, patient safety was maintained while working with pharmacist independent prescribers [40]. In a systematic literature review, Walpola et al. analysed the accessibility of pharmacist prescribing and the impact on access to medicine. Further, pharmacists involved in prescribing have a positive impact on access to medicine [29]. Participants agreed that access to medicines for minor illnesses or repeat prescriptions could be facilitated by the widespread and convenient location of pharmacies.

In addition, there was a broad consensus among the participants in this study: the introduction of pharmacist prescribing could help relieve the burden on German GP practices. The extent to which GP practices could be relieved depended on the pharmacist’s prescribing model. Pillay et al. showed the personnel and financial efforts required in GP practices in the UK to care for minor ailments. For example, 90% of all examinations treated minor ailments. This correlated with 20% of the workload in GP practices [41]. Thus, CPs, if permitted to prescribe medication for minor ailments within a defined framework, could have a great potential to reduce the workload of GP practices in Germany.

Herein, there was a strong agreement among participants (94.6%) in favour of health insurance companies covering medicines prescribed by pharmacists, suggesting that the frequency of future use of prescription services provided by CPs may depend on whether the possibility of reimbursement for pharmacist-prescribed drugs by health insurance companies exists. Moreover, the economic feasibility of health insurance companies to cover the costs of medicines prescribed by pharmacists must be analysed further from a health economic perspective. Nevertheless, there was evidence of the economic viability of pharmacists’ prescribing practises [42].

To take on a potential prescribing role, most participants believed that the clinical assessment skills of pharmacists need to be enhanced and that further training or education, adapted to the level of prescribing authorisation, should be mandatory. Other studies confirmed the need to improve the clinical skills of pharmacists [43,44]. In particular, Scotland is putting a lot of effort into training to increase the number of independent prescribers to maintain and expand its ‘Pharmacy First Plus’ programme [45].

Thus, there was considerable agreement among participants regarding their assessment of various aspects concerning pharmacist prescribing. A key finding of this research was the notably open and positive attitude of participants towards pharmacist prescribing. Even when asked whether participants could imagine the introduction of pharmacist prescribing in Germany, most participants (88.0%) agreed. The prioritisation of advice quality over the professional background of the adviser by participants suggested receptiveness to innovation, reflecting progressive rather than entrenched perspectives. Gerard et al. surveyed patients in various GP practices and demonstrated a similar openness to pharmacists prescribing medications. The questionnaire assessed patients’ general openness towards existing prescriptions by pharmacists, concluding that pharmacists’ services were valued by patients and represented an acceptable form of service provision [46].

To evaluate whether the results of this research are representative of the overall pharmacy customer population, we compared our study participants with those from the representative Forsa survey [7] conducted in March 2021 on behalf of the Federal Union of German Associations of Pharmacists (ABDA), which included German citizens aged 18 and older. Across all age groups, the proportion of participants who permanently took three or more prescription drugs was 18.5% in the Forsa survey, compared to 21.5% in this study. Within the 50–69 age group, 22.98% of our participants reported taking three or more prescription drugs, a proportion comparable to the 24.96% observed in the Forsa survey. For those aged ≥70 years, the percentage in our study was remarkably higher at 72.1%, compared with 41.25% in the Forsa survey [7]. The gender distribution analysis revealed a clear overrepresentation of the female gender, in comparison to the general pharmacy customer base in Germany and the population of Baden-Württemberg aged ≥18 years [35].

Research indicates that women in Germany utilise health services more often than men, a trend observed in rural populations in one study and urban populations in another [47,48]. Additionally, ‘the use of medicines, both prescribed and self-medicated, is also higher among women than among men’, according to the Robert Koch Institute [49]. The increased utilisation of healthcare professionals, coupled with the higher consumption of medications among women, may influence their interactions with pharmacies. This trend could potentially elucidate the observed gender differences in survey participation.

### Strengths and limitations

Pharmacist prescribing is a novel approach, given that it has not yet been introduced in Germany. This study provides valuable insights into the potential of implementing pharmacist prescribing and contributes to further discourse on this innovation. However, this study predominantly focused on the views of pharmacy customers regarding pharmacist prescribing and its potential benefits. We recognise that a comprehensive evaluation of pharmacist prescribing would also need to consider concerns, risks, practical challenges and regulatory barriers associated with its implementation. Our study was only able to address these wider considerations to a limited degree. This focus on pharmacy customer opinions might lead to a more positive overall impression.

The survey was primarily distributed to participants at the pharmacies. This broad distribution might have contributed to a low response rate, which represents a limitation and could have introduced a bias. Using convenience sampling inherently carries limitations. Given that participants were recruited from community pharmacies and the survey link was also shared on their public Facebook© pages, individuals may already have held more positive perceptions of pharmacists. This could have resulted in more favourable responses compared with the general population. Consequently, this sampling method may have led to selection bias. Participation partially relied on self-enrolment, but also involved pharmacy staff actively drawing customers’ attention to the study and inviting them to the designated stand for more information. This dual approach may introduce a selection bias, as individuals with a greater interest in the subject matter might have been more inclined to self-enrol and participate. While the survey link was shared on the public Facebook© pages of the five community pharmacies, this does not ensure that all participants were customers of these pharmacies. Assumably, most followers of these pages potentially reside in Baden-Württemberg. Consequently, the comparison made with Baden-Württemberg’s demographics may have limited validity. However, assuming that most participants were from this region, we considered this comparison appropriate. The comparison of the study participants with those in the Forsa survey [7] and the overall population of Baden-Württemberg also indicates a bias. The average age of the study participants, at 51.04 years, is comparable to that at 50.75 years for residents of Baden-Württemberg aged ≥18 years [35]. Furthermore, the transferability of the data to other countries may be limited, given the survey was conducted in Germany. The research is based on survey data; therefore, there could be a bias in the responses. However, upon evaluating the questionnaires, no evidence of such a bias was detected.

### Recommendations for further research

Future research should include pharmacists as the primary target group, in addition to other stakeholders in healthcare such as doctors, to gain a comprehensive understanding of pharmacist prescribing; this could be achieved through survey studies or qualitative research using semi-structured interviews, which would provide detailed thematic analysis and insights. A successful cooperation can only be achieved if all decision makers are consulted and their interests are considered. Further research is needed to address the practical challenges and regulatory barriers affecting the potential introduction and to assess the feasibility of pharmacist prescribing in practise. In addition, defining the educational objectives and measures of pharmacists is crucial for the potential introduction. To enhance the public acceptance of a potential introduction of pharmacist prescribing, investigating the reasons for negative views or objections towards pharmacist prescribing is important. Therefore, further research should include a detailed analysis of these concerns.

## Conclusion

Participants, regardless of age and gender, were open and positive about pharmacists prescribing and supportive of its potential implementation in Germany in this setting. The identified high level of participants trust in pharmacists provides a foundation for potentially expanding the role of pharmacists. Moreover, participants acknowledged the importance of pharmacists undergoing training before being authorised. To enhance practical feasibility, further research is required to incorporate the CP and other stakeholders’ perspectives regarding pharmacist prescribing.
